# *Brassica napus* Transcription Factor *Bna.A07.WRKY70* Negatively Regulates Leaf Senescence in *Arabidopsis thaliana*

**DOI:** 10.3390/plants12020347

**Published:** 2023-01-11

**Authors:** Tiantian Liu, Yuxin Li, Chang Wang, Da Zhang, Jiajia Liu, Mingyuan He, Mingxun Chen, Yuan Guo

**Affiliations:** National Yangling Agricultural Biotechnology & Breeding Center, Shaanxi Key Laboratory of Crop Heterosis, and College of Agronomy, Northwest A&F University, Yangling 712100, China

**Keywords:** *Bna.A07.WRKY70*, leaf senescence, *Arabidopsis thaliana*, *Brassica napus*

## Abstract

Leaf senescence is the final stage of leaf development and is essential for storage properties and crop productivity. WRKY transcription factors have been revealed to play crucial roles in several biological processes during plant growth and development, especially in leaf senescence. However, the functions of *Brassica napus* WRKY transcription factors in leaf senescence remain unclear. In the present study, *Bna.A07.WRKY70*, one paralogue of *Brassica napus WRKY70*, was cloned from the *B. napus* cultivar “Zhongshuang11 (ZS11)”. We found that Bna.A07.WRKY70 contains a highly conserved WRKY domain and is most closely related to *Arabidopsis thaliana* WRKY70. The subcellular localization and transcriptional self-activation assays indicated that Bna.A07.WRKY70 functions as a transcription factor. Meanwhile, RT-qPCR and promoter-GUS analysis showed that *Bna.A07.WRKY70* is predominantly expressed in the leaves of *B. napus* and rosette leaves of *A. thaliana*. In addition, our results demonstrated that ectopic expression of *Bna.A07.WRKY70* in *A. thaliana wrky70* mutants could restore the senescence phenotypes to wild-type levels. Consistently, the expression levels of three senescence-related marker genes of *wrky70* mutants were restored to wild-type levels by ectopic expression of *Bna.A07.WRKY70*. These findings improve our understanding of the function of *Bna.A07.WRKY70* in *B. napus* and provide a novel strategy for breeding the new stay-green cultivars in rapeseed through genetic manipulation.

## 1. Introduction

Rapeseed (*Brassica napus* L., AACC, 2n = 38), an allotetraploid species, originated from spontaneous hybridization between two diploid *Brassica* species: *Brassica rapa* (AA, 2n = 20) and *Brassica oleracea* (CC, 2n = 18) [1]. It is a major oilseed crop grown worldwide for the production of edible oil in the human diet, livestock feed, and industrial materials [2]. Therefore, there is important social and economic significance for studying its associated biological processes, including leaf development. The leaf is the primary organ of photosynthesis and can produce nutrition and gather energy during plants’ growth and maturation stages. Leaf senescence, as a type of programmed cell death (PCD), is the terminal stage of leaf development. During leaf senescence, the chloroplast first starts disassembling, and is followed by a loss of chlorophyll together with the catabolism of macromolecules such as protein, lipids, nucleic acids, and RNA [3]. By general catabolism, cellular materials are converted into easily exportable nutrients, which from senescing leaves were subsequently transported to reproductive and developing structures [4]. Consequently, leaf senescence is a critical process for crop fitness and is particularly essential for the optimization of crop productivity. Generally, leaf senescence is influenced by various external environmental and endogenous factors. The environmental cues that affect leaf senescence include high temperature, light signals, drought, and biotic stress [5,6,7]. The endogenous factors include the accumulation of reactive oxygen species (ROS), variation of plant hormones, and, most importantly, regulation of multiple senescence-associated genes [8,9,10,11]. Therefore, mining the key genes regulating the leaf senescence process is of great importance in rapeseed.

The WRKY proteins are one of the largest and most important superfamilies of transcription factors (TFs) in plants. WRKY transcription factors encompass a core motif WRKYGQK (a highly conserved WRKY domain) at the N-terminus and an atypical Zinc finger motif at the C-terminus [12]. On the basis of both the number of WRKY domains and the features of the Zn-finger motif in their evolutionary history, the WRKY TFs can be divided into three different groups (I, II, and III). Group I contains two WRKY domains and a finger motif whose pattern is conserved zinc ligands (C–X_4-5_–C–X_22-23_–H–X_1_–H), which is the same as the Zinc finger motif of group II, but there is only one WRKY domain in group II. Instead of a C2–H2 pattern in group I and II, group III contains a pattern of C2–HC (C–X_7_–C–X_23_–H–X_1_–C) zinc finger-like motif and have one WRKY domain [13,14]. All three groups’ members of WRKY TFs have been demonstrated to interact with the specific DNA *cis*-acting element W-box (C/TTGACT/C) in the promoter regions of downstream genes and further regulate their expression [15]. In recent decades, experimental evidence has shown that WRKY proteins act as key regulators widely involved in various plant growth and development processes, such as leaf senescence, growth of roots [16], stem elongation [17], and multiple biotic and abiotic stressors [18,19]. In *Arabidopsis thaliana*, *WRKY53* acts in a complex transcription factor signaling network regulating leaf senescence-specific gene expression [20], WRKY45 interacts with the DELLA protein RGL1 to positively regulate age-triggered leaf senescence [21], and *WRKY71* mediates ethylene (ET) signaling and synthesis to hasten leaf senescence [22].

*WRKY70* belongs to WRKY III TFs and has been reported in response to several developmental and physiological processes in diversified species [23]. In *Arabidopsis*, *WRKY70* acts as a negative regulator of leaf senescence, with gradually increasing expression during leaf development [3], and *WRKY70* is also crucial in plant defense against pathogens, controlling the cross-talk of salicylic acid (SA) and jasmonic acid (JA) signaling in plant defense [23,24]. Moreover, *WRKY70* is an important signaling component that is positively involved in brassinolide (BR)-regulated growth and negatively involved in drought responses by inhibiting drought-responsive genes [25]. In chickpeas, *WRKY70* was reported to regulate the expression of a chickpea HD-Zip transcription factor *CaHDZ12*, which improved tolerance to osmotic stresses under drought and salinity stress, and increased sensitivity to abscisic acid (ABA) in transgenic tobacco and chickpea [26]. In addition, it was suggested that GhWRKY70D13 negatively regulates cotton’s resistance to *Verticillium dahliae* mainly through its effect on ET and JA biosynthesis and signaling pathways [27]. A recent study demonstrated that *TaWRKY70* positively regulates *TaCAT5* by directly binding to the *TaCAT5* promoter to enhance Cd tolerance in transgenic *Arabidopsis* [28]. In *B. napus*, the *BnWRKY70* knockout plants by CRISPR/Cas9 system enhanced *Sclerotinia sclerotiorum* resistances, while overexpression of *BnWRKY70* reduced resistance to *S. sclerotiorum* [29]. However, the roles of WRKY proteins in *B. napus* in the regulation of leaf senescence remain unclear.

In the current study, *Bna.A07.WRKY70*, one of the *AtWRKY70* orthologues in *B. napus*, was isolated and functionally characterized. We found that *Bna.A07.WRKY70* functioned as a TF and was specifically expressed in the leaves in *A. thaliana* and *B. napus*. We also demonstrated that ectopic expression of *Bna.A07.WRKY70* in the *A. thaliana wrky70* mutant restored the leaf senescence rate and chlorophyll content and greatly altered the expression of three senescence-related genes in this mutant. Our results may indicate that *Bna.A07.WRKY70* functions as a negative regulator of leaf senescence in *Arabidopsis*, which might reveal a conserved role of WRKY70 proteins in regulating leaf senescence between *A. thaliana* and *B. napus*.

## 2. Results

### 2.1. Sequence Analysis of BnaWRKY70 Paralogs

**Figure 1:**
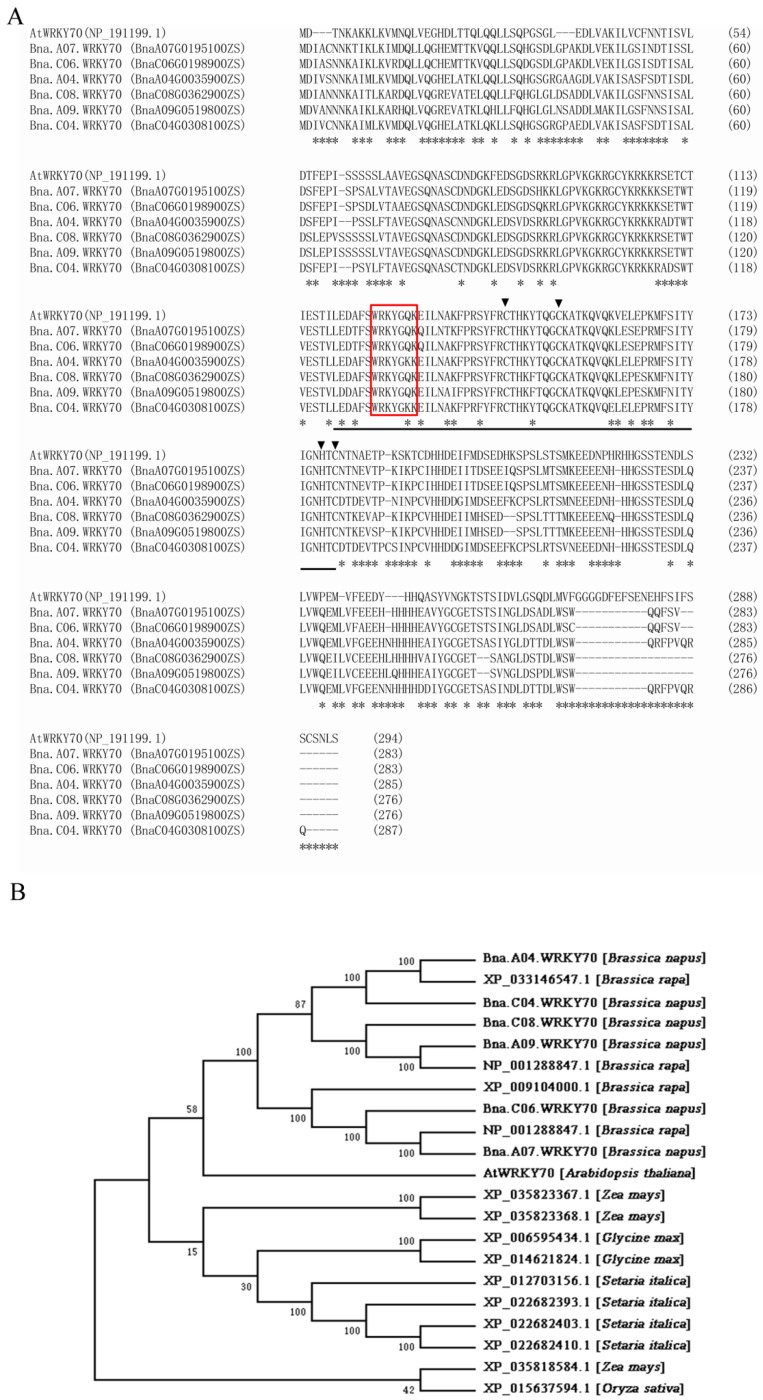
Protein sequence and phylogenetic analyses of WRKY70 proteins. (**A**) Protein sequence alignment of WRKY70 from *A. thaliana* and *B. napus* was carried out using the MUSCLE program (http://www.ebi.ac.uk/Tools/msa/muscle/, accessed on 12 September 2022). Asterisks indicate non-conservative differences. The WRKY domain 125–185, as indicated by the Conserved Domain Search program (http://www.ncbi.nlm.nih.gov/Structure/cdd/wrpsb.cgi, accessed on 12 September 2022), is underlined. The highly conserved core sequence WRKYGQK in the WRKY domain is represented by a red box, together with the C and H residues in the CCHC zinc-finger-like motif indicated by a downward black triangle. (**B**) Phylogenetic analysis of Bna.A07.WRKY70 with 20 other WRKY70 proteins from seven plant species, including AtWRKY70 (*Arabidopsis thaliana*); Bna.A07.WRKY70 (BnaA07G0195100ZS), Bna.C06.WRKY70 (BnaC06G0198900ZS), Bna.A04.WRKY70 (BnaA04G0035900ZS), Bna.C08.WRKY70 (BnaC08G0362900ZS), Bna.A09.WRKY70 (BnaA09G0519800ZS), and Bna.C04.WRKY70 (BnaC04G0308100ZS (*Brassica napus*); NP_001288821.1, XP_033146547.1, XP_009104000.1, and NP_001288847.1 (*Brassica rapa*); XP_015637594.1 (*Oryza sativa*); XP_035823367.1, XP_035823368.1, and XP_035818584.1 (*Zea mays*); XP_012703156.1, XP_022682393.1, XP_022682403.1, and XP_022682410.1 (*Setaria italica*) and XP_006595434.1 and XP_014621824.1 (*Glycine max*). A neighbor-joining tree (Jones–Taylor–Thornton model) with 1000 replicates of bootstrap analysis was generated by MEGA7. Bootstrap values are indicated at the nodes, and the accession numbers of the species are labeled on the phylogenetic tree.

In the *B. napus* cultivar “Zhongshuang11 (ZS11)”, six paralogs of BnaWRKY70 were predicted in BnPIR (http://cbi.hzau.edu.cn/bnapus/index.php, accessed on 9 September 2022) and were designated Bna.A07.WRKY70 (BnaA07G0195100ZS), Bna.C06.WRKY70 (BnaC06G0198900ZS), Bna.A04.WRKY70 (BnaA04G0035900ZS), Bna.C08.WRKY70 (BnaC08G0362900ZS), Bna.A09.WRKY70 (BnaA09G0519800ZS), and Bna.C04.WRKY70 (BnaC04G0308100ZS). With the multiple sequence alignment, we found that the WRKY70 protein from *B. napus* and *A. thaliana* possessed highly conserved WRKY domains, including WRKYGQ/KK core motif and a pattern of C2–HC zinc finger-like motif at the C-terminus (Figure 1A). Among them, Bna.A07.WRKY70 was predicted to share the highest identity in the amino acid sequence with the AtWRKY70 protein (66.01%) (Figure S1). A phylogenetic analysis was performed to investigate the evolutionary relationships between Bna.A07.WRKY70 and 20 WRKY70 proteins from seven plant species, including *A*. *thaliana*, *B*. *napus*, *B*. *rapa*, *Glycine max*, *Zea mays*, *Oryza sativa*, and *Setaria italic*. As illustrated in Figure 1B, Bna.A07.WRKY70 is most closely related to the WRKY70 protein from *B. rapa* (NP_001288847.1) and *A. thaliana* (AtWRKY70). These results suggested preliminarily that Bna.A07.WRKY70 might have similar functions as AtWRKY70.

### 2.2. Subcellular Localization and Transcriptional Activity of Bna.A07.WRKY70

**Figure 2:**
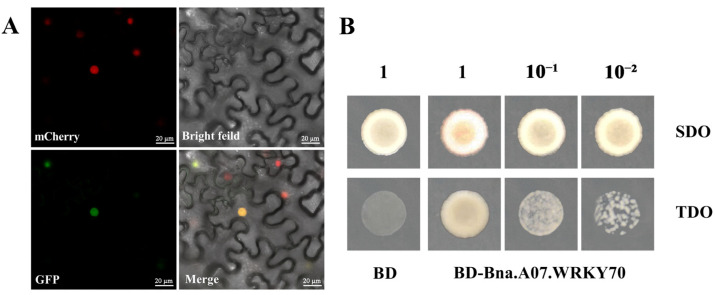
Transcription factor characterization of Bna.A07.WRKY70. (**A**) Subcellular localization of Bna.A07.WRKY70 protein fused with GFP (*35S:GFP-Bna.A07.WRKY70*) in tobacco leaves (*Nicotiana benthamiana*). mCherry, a nuclear-localized protein fused with a red fluorescent protein; merge, merge of mCherry, GFP, and bright field images. (**B**) Transcriptional activation assays of Bna.A07.WRKY70 in yeast. BD: empty vector that contains GAL4 DNA-binding domain, *BD-Bna.A07.WRKY70*: cDNAs encoding of *Bna.A07.WRKY70* transcripts were separately cloned into the pGBKT7/BD vector containing the GAL4 DNA binding domain, which transformed into the yeast strain Y2HGold, SDO: ability of yeast transformants to grow on medium lacking Trp, TDO: ability of yeast transformants to grow on medium lacking Trp, His and Ade indicates transcriptional activation. 1, 10^−1^, 10^−2^: the transformed strains were spotted on plates by diluting to different concentrations. The images show representative results from more than four independent yeast transformants.

For subcellular localization, Bna.A07.WRKY70 was expressed in tobacco (*Nicotiana benthamiana*) leaf cells as a recombinant protein fused to a green fluorescent protein marker. The fluorescence signal was detected in the nucleus by laser scanning confocal microscopy (Figure 2A), suggesting that Bna.A07.WRKY70 might function as a transcription factor.

To further characterize Bna.A07.WRKY70 function, we investigated whether Bna.A07.WRKY70 has transcription activation activity in yeast cells. The empty vector pGBKT7 as negative control and fusion construct (*pBD-Bna.A07.WRKY70*) were transformed separately into Y2HGold yeast cells, which were cultured on SDO (SD/-Trp) and TDO (SD/-Trp/-His/-Ade) medium. As shown in Figure 2B, on SDO (SD/-Trp) medium, all yeast transformants could grow normally, indicating that the constructs were transformed successfully into the Y2HGold yeast cells. Instead, on TDO (SD/-Trp/-His/-Ade) medium, the empty vector pGBKT7 did not grow, but yeast cells with Bna.A07.WRKY70 fusion constructs grew well, which demonstrated that Bna.A07.WRKY70 could activate the expression of the reporter genes. Given these findings, the Bna.A07.WRKY70 was testified to function as a transcription activator.

### 2.3. Analysis of Bna.A07.WRKY70 Expression Pattern

**Figure 3:**
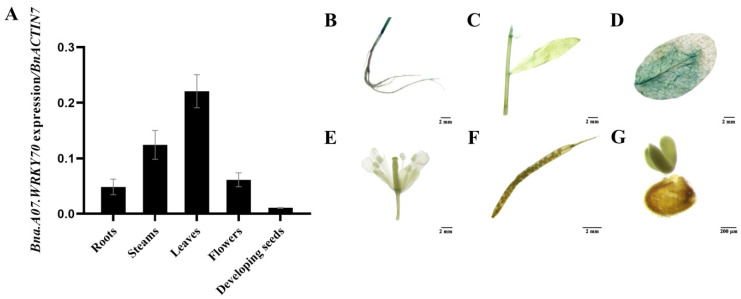
Analysis of the *Bna.A07.WRKY70* expression pattern. (**A**) RT-qPCR analysis of the *Bna.A07.WRKY70* expression in various tissues of *B. napus* cultivar “ZS11”. The RT-qPCR result was normalized against the expression of *BnACTIN7* as an internal control. Values are means ± SD (n = 3). Error bars denote standard deviations. (**B**) to (**G**), Histochemical GUS staining in 35-day-old *ProBna.A07.WRKY70:GUS* transgenic *Arabidopsis* plants. (**B**) Roots (bar = 2 mM); (**C**) Stems and leaves (bar = 2 mM); (**D**) rosette leaves (bar = 2 mM); (**E**) Flowers (bar = 2 mM); (**F**) siliques 12 days after pollination (bar = 2 mM); (**G**) Developing seeds 12 days after pollination (bar = 200 μM).

We further investigated the spatiotemporal expression pattern of *Bna.A07.WRKY70* by analyzing the relative abundance of the mRNA in various tissues of *B. napus* cultivar “ZS11” using quantitative reverse transcription PCR (RT-qPCR). The results showed that *Bna.A07.WRKY70* was widely expressed in different organs of *B. napus*, with higher expression in leaves, moderate in stems, flowers, and roots but low in developing seeds (Figure 3A). To comprehensively investigate the spatiotemporal expression pattern of *Bna.A07.WRKY70* in *A. thaliana*, we obtained 16 *pBna.A07.WRKY70:GUS* in wild-type background independent lines of *A. thaliana* and the one representative line was used for promoter-GUS analysis because of similar GUS staining patterns in most lines. Consistent with the RT-qPCR data in *B. napus*, promoter-GUS activity staining was predominantly detected in rosette leaves of *A. thaliana* (Figure 3D) and then was also slightly detected in other organs of *A. thaliana*, including stems (Figure 3C), roots (Figure 3B), and flower abscission zones (Figure 3E). Conversely, it was not detected in the embryo (Figure 3G) and siliques (Figure 3F). In summary, these observations suggested that *Bna.A07.WRKY70* might regulate a significant function during leaf development.

### 2.4. Bna.A07.WRKY70 Negatively Regulates Leaf Senescence in A. thaliana

**Figure 4:**
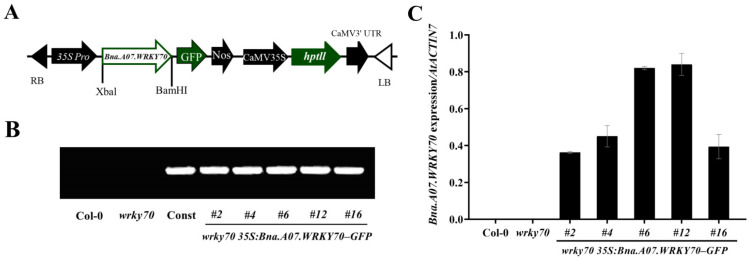
Molecular characterization of *wrky70 35S:Bna.A07.WRKY70*-*GFP* transgenic plants. (**A**) Schematic diagram of constitutive expression cassette of the *Bna.A07.WRKY70* gene in the binary vector pCAMBIA-1300 used for plant transformation. RB, right border; LB, left border; 35S Pro, CaMV 35S promoter; Nos, nopaline synthase terminator; CaMV35S, CaMV 35S promoter; *hptII*, hygromycin resistance gene. (**B**) PCR-based DNA genotyping of *wrky70 35S:Bna.A07.WRKY70*-*GFP* transgenic plants using specific primers of 35S_P/Bna.A07.WRKY70-GFP-BamHI-R. Const, *35S:Bna.A07.WRKY70*-*GFP* construct. Col-0 and *wrky70* indicate *A. thaliana* wild type and mutant plants, respectively. (**C**) Expression analysis of *Bna.A07.WRKY70* in *wrky70 35S:Bna.A07.WRKY70*-*GFP* transgenic plants using RT-qPCR. The expression level was normalized against the expression of *AtACTIN7*, which was used as an internal control. Values are the means ± SD (n = 3). Error bars indicate standard deviation. # indicates the transgenic lines.

**Figure 5:**
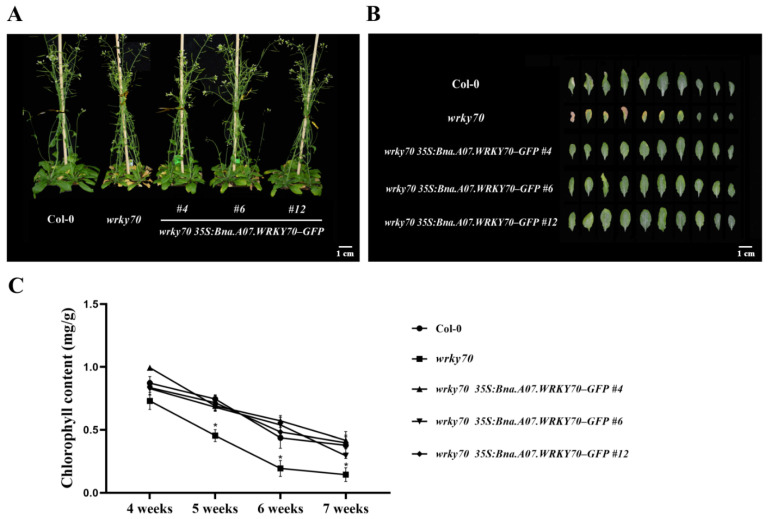
Effects of *Bna.A07.WRKY70* overexpression in the *wrky70* mutant background on leaf senescence in *A. thaliana*. (**A**) The whole plant phenotypes of leaf senescence in the wild type (Col-0), *wrky70* mutant, and *wrky70 35S:Bna.A07.WRKY70-GFP* transgenic plants. The images were taken 35 days after germination (DAG). Bar = 1 cM. (**B**) Phenotype of rosette leaves in 35-day-old plants, excised leaves are arranged according to age, from older to younger. Bar = 1 cM. (**C**) Comparisons of chlorophyll content of the fifth to sixth rosette leaves among wild-type (Col-0), *wrky70* mutant, and *wrky70 35S:Bna.A07.WRKY70* transgenic plants at the indicated ages. Values are means ± SD (n = 3). Asterisks indicate significant differences from wild-type (two-tailed paired Student’s *t*-test, *p* ≤ 0.05). Error bars indicate standard deviation. # indicates the transgenic lines.

To further explore the function of *Bna.A07.WRKY70* on leaf development, we introduced the construct *35S:Bna.A07.WRKY70-GFP* into *A. thaliana wrky70* mutant (Figure 4A). Twelve independent T_1_ transgenic plants were generated using hygromycin selection, and five independent T_3_ homozygous transgenic lines *wrky70 35S:Bna.A07.WRKY70-GFP* were selected and confirmed by PCR amplification with the specific primers 35S-F/Bna.A07.WRKY70-GFP-BamHI-R (Figure 4B; Supplementary Table S1). Of these lines, three representative ones, *wrky70 35S:Bna.A07.WRKY70-GFP #4*, *#6*, and *#12*, with a relatively high expression level (Figure 4C), were selected for the follow-up experiment. As illustrated in Figure 5A,B, the loss-of-function mutants *wrky70* exhibited markedly yellowing of leaves at 35 DAG (days after germination) and indicated earlier senescence compared to wild-type plants, which is in line with previous findings [3]. Interestingly, we found that the *A. thaliana wrky70* mutant leaves were smaller than wild-type plants. Ectopic expression of *Bna.A07.WRKY70* fully restored the rate of leaf senescence to wild-type levels in *Arabidopsis wrky70* mutants (Figure 5A). Furthermore, by arranging the rosette leaves of 35-day-old Col-0, *wrky70* mutant, and transgenic plants (*#4*, *#6*, *#12*) according to their age from older to younger, we found that three *Bna.A07.WRKY70* transgenic lines in the *wrky70* background delayed the premature senescence of leaves and rescued the phenotype of leaves relatively smaller in size compared to wild-type plants (Figure 5B). The results of the chlorophyll content indicated that the chlorophyll content of the *wrky70* mutant intensified degradation from the fifth week, but the chlorophyll content of *Bna.A07.WRKY70* transgenic lines were in keeping with that of Col-0 and clearly higher than that of the *wrky70* mutant (Figure 5C).

**Figure 6:**
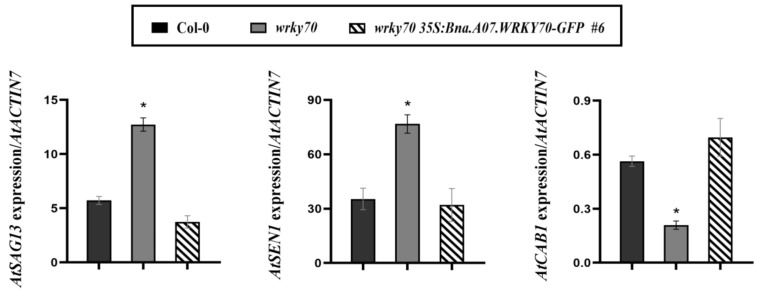
Expression analysis of leaf senescence marker genes in the rosette leaves among wild-type (Col-0), *wrky70* mutant, and *wrky70 35S:Bna.A07.WRKY70-GFP* transgenic plants at the 35 DAG, as measured by RT-qPCR. Expression levels were normalized to the expression of the internal reference gene, *AtACTIN7*. Values are means ± SD (n = 3). Error bars indicate standard deviations. Asterisks indicate statistically significant differences from wild type plants (two-tailed paired Student’s *t*-test, *p* ≤ 0.05). # indicates the transgenic lines.

In order to further confirm that the *Bna.A07.WRKY70* regulates the progress of leaf senescence in *A. thaliana*, we assessed the transcript levels of representative genes relating to senescence in the fifth and sixth rosette leaves of *A. thaliana* wild-type, the *wrky70* mutant, *wrky70 35S:Bna.A07.WRKY70-GFP* plants at 35 DAG. Compared to the wild type, the results showed that the expression of the senescence-related gene *AtSAG13* (*senescence-associated gene 13*) and *AtSEN1* (*senescence-associated gene 1*) were significantly increased, while the expression of photosynthesis-related *AtCAB1* gene (*chlorophyll a/b-binding protein*) was significantly decreased in 35-day-old *wrky70* mutant plants (Figure 6). However, when the *35S:Bna.A07.WRKY70-GFP* was introduced into the *wrky70* mutant, we found that the transcript abundance of these three senescence-related marker genes, including *AtSEN1*, *AtCAB1*, and *AtSAG13*, was restored to wild type levels. In brief, all results containing the premature senescence phenotype, chlorophyll content, and the expression of senescence-associated marker genes together revealed that *Bna.A07.WRKY70* may negatively regulate the leaf senescence by adjusting the expression of senescence genes in *A. thaliana* and play a similar role with *AtWRKY70* in *A. thaliana*.

## 3. Discussion

Leaf senescence is an indispensable portion and spans the latter half of leaf development. It is a highly intricate process regulated by multiple pathways [30]. As previously reported, the three largest groups of transcription factors, WRKY, NAC, and MYB superfamilies, are responsible for modulating transcriptional changes during leaf senescence [31], in which the *AtWRKY70* has already been confirmed with a high level of expression in the late stage of leaf development and functions as an essential repressor during leaf senescence in *A. thaliana* [32]. However, the roles of *WRKY70* transcription factors during leaf development in *B. napus* were lacking.

It has been widely known that *B. napus* was formed 7500 years ago by natural hybridization between *B. rapa* and *B. oleracea* [33]. *B. napus*, and diploid parental species *B. rapa* and *B. oleracea*, are believed to share a common ancestor with *A. thaliana*, a fact that has favored the transfer of knowledge from *Arabidopsis* to *B*. *napus*. As an allopolyploid plant, a large number and a high frequency of chromosome variation activities were identified, such as duplication, rearrangement, fusion, and deletion in the evolution processes of *B. napus*, which makes the genomics of *B*. *napus* more complicated. Generally, a single *Arabidopsis* gene is represented by two to eight paralogs in the *B. napus* genome [34,35]. Accordingly, six paralogues (Bna.A07.WRKY70, Bna.C06.WRKY70, Bna.A04.WRKY70, Bna.C08.WRKY70, Bna.A09.WRKY70, and Bna.C04.WRKY70) were found in the *B. napus* genome (Figure 1). In the WRKY transcription factor family, the WRKY domain is the major determinant of DNA-binding and specifically binds DNA *cis*-acting element W-box (C/TTGACT/C). Our results showed that all six Bna.WRKY70 had the WRKY protein domain containing the WRKYGQ/KK core motif and a pattern of C2–HC zinc finger-like motif at the C-terminus (Figure 1A). In the present study, among these BnaWRKY70 paralogues, Bna.A07.WRKY70, which was predicted to have the highest identity of protein sequence and the WRKY central conserved domains with AtWRKY70 (Figure 1), was cloned from the *B. napus* cultivar “ZS11” and functionally characterized. Bna.A07.WRKY70 was located in the nucleus of tobacco leaf cells, and we further demonstrated that Bna.A07.WRKY70 could activate the expression of the reporter genes in yeast cells (Figure 2). These results suggested that Bna.A07.WRKY70 functions as a transcription activator. Additionally, the *Bna.A07.WRKY70* transcript was broadly present in different vegetative tissues, with the highest levels observed in leaves (Figure 3), suggesting that *Bna.A07.WRKY70* might regulate a significant function during leaf development. Ectopic expression of *Bna.A07.WRKY70* in the background of *A. thaliana wrky70* mutants significantly delayed the senescence of leaves and restored the chlorophyll content to the wild type level (Figure 5). Moreover, the expression of senescence-associated genes (*AtSEN1*, *AtSAG13*, and *AtCAB1*) was clearly regulated by *Bna.A07.WRKY70* during leaf senescence. Thus, these results may indicate that *Bna.A07.WRKY70* functions as a negative factor in leaf senescence as the *AtWRKY70*.

During leaf senescence, the leaves turned yellow, resulting in photosynthesis deficiency and beginning with chloroplast dismantling, followed by degradation of chlorophyll and chlorophyll-protein complexes. Meanwhile, leaf senescence is accompanied by decreased expression of genes related to photosynthesis and protein synthesis and increased expression of senescence-associated genes (SAGs) [36]. Consistently, our results demonstrated that compared to the wild type, the expression of the photosynthesis-related *AtCAB1* gene was decreased, and the expression of senescence-related gene *AtSAG13* and *AtSEN1* were increased in *A. thaliana wrky70* mutant plants. The expression of these three marker genes was rescued to wild-type in *wrky70 35S:Bna.A07.WRKY70-GFP* transgenic plants, which proved that *Bna.A07.WRKY70* indeed delayed the leaf senescence during plant senescence by affecting the expression of these three senescence genes in *A. thaliana*. Leaf senescence was widely influenced by a variety of external and internal factors, including environmental stresses and phytohormones. Recently, key gene regulatory networks comprising these TFs have been identified, indicating that leaf senescence is controlled by multiple cross-linking pathways, many of which are associated with stress response signaling [37,38,39]. *Arabidopsis WRKY71* was reported that it is able to directly upregulate the ethylene signaling pathway genes *EIN2* (*ethylene insensitive2*) and *ORE1* (*oresara1*) and promote ethylene synthesis by directly activating the *ACS2* gene to accelerate leaf senescence in *Arabidopsis* [22]. The cotton (*Gossypium hirsutum* L.) *GhWRKY91* directly targets *GhWRKY17*, a gene associated with ABA signals and reactive oxygen species (ROS) production to negatively mediate leaf senescence and provide a foundation for further functional studies on natural and stress-induced leaf senescence [40]. *OsWRKY53* of rice, as a positive regulator, repressed the transcript of ABA catabolic genes (*OsABA8ox1* and *OsABA8ox2*) by directly binding to their promoters to promote ABA accumulation, and modulated ABA-induced leaf senescence [41]. In *Arabidopsis*, *AtWRKY70* transcript levels were more strongly reduced in *npr1* (*non-expressor of PR 1*) and *pad4* (*phytoalexin-deficient 4*) and completely abolished in *NahG* (salicylate hydroxylase gene) plants compared to wild-type at 40 days post germination, among which, the *NahG*, *pad4*, and *npr1* belonged to SA mutants and exhibited a delayed senescence phenotype [3]. These findings support the role of *AtWRKY70* as a senescence-associated gene and indicate a functional requirement of SA for its normal expression. Besides, the preceding research illustrated that the pathway of plant hormones could respond to numerous abiotic stresses; for instance, *GhWRKY17* from upland cotton modulated the increased sensitivity of plants to drought by reducing the level of ABA, and repressed transcript levels of ABA-inducible genes, including *AREB* (*ABA-responsive element binding*), *DREB* (*dehydration-responsive element binding*), *NCED* (*9-cis-epoxycarotenoid dioxygenase*), *ERD* (*early responsive to dehydration*) and *LEA* (*late embryogenesis-abundant protein*) under drought and salt stress conditions, indicating that *GhWRKY17* responds to drought and salt stress through ABA signaling and the regulation of cellular ROS production in plants [42]. With the above findings in mind, whether *Bna.A07.WRKY70* of *B. napus* adjusts the signaling pathways of phytohormone by combining with some key genes during the regulation of leaf senescence and responds to plant stress resistance mediated by the signaling pathways, can be explored further.

Interestingly, it has been reported that the *Arabidopsis wrky70* knockout mutants were slightly reduced in size compared to wild-type plants during the entire period of development in *A. thaliana* [3]. However, from another report, no obvious growth phenotype was observed in a single knockout mutant of *wrky70* compared with the wild-type *A. thaliana* [25]. In this study, our results found that the *A. thaliana wrky70* mutant leaves were smaller than wild-type plants, and the leaf size of the *wrky70* mutant was restored to wild type by the ectopic expression of *Bna.A07.WRKY70*. Whether or not *Bna.A07.WRKY70* plays a role in controlling the size of leaves requires further investigation. Overall, based on the above, the multiple functions and regulation network of *Bna.A07.WRKY70* still has great research potential.

## 4. Materials and Methods

### 4.1. Plant Materials and Growth Conditions

The *A. thaliana* wild-type ecotype Columbia (Col-0), the T-DNA mutant *wrky70* (SALK_025198) in the Col-0 background obtained from Arashare (https://www.arashare.cn/index/, accessed on 20 October 2020), and *Brassica napus* L. cultivar “Zhongshuang 11 (ZS11)”, were used in this study. The *A. thaliana* plants were grown in a growth chamber at 22 °C under a long day duration (LD, 16 h light/8 h dark) with moderate light intensity (160 μmol m^−2^ s^−1^). The *B. napus* cultivar “ZS11” was first grown in the greenhouse at 22 °C with a long day duration for six weeks. For vernalization, the plants were transferred to a cold chamber at 4 °C under LD conditions. After vernalization, the plants were returned to the initial greenhouse conditions for 10 weeks until harvest.

### 4.2. Protein Sequence and Phylogenetic Analysis

The protein sequences of WRKY70 were obtained from the National Center for Biotechnology Information (NCBI) database (https://www.ncbi.nlm.nih.gov/, accessed on 9 September 2022) and the *B. napus* pan-genome information resource (BnPIR) database (http://cbi.hzau.edu.cn/bnapus/index.php, accessed on 9 September 2022). Protein sequence alignment was carried out using MUSCLE (http://www.ebi.ac.uk/Tools/msa/muscle/, accessed on 12 September 2022). The conserved WRKY domain of Bna.A07.WRKY70 was indicated using the conserved domain search program in the National Center for Biotechnology Information (http://www.ncbi.nlm.nih.gov/Structure/cdd/wrpsb.cgi, accessed on 12 September 2022). The phylogenetic tree was conducted using the neighbor-joining tree (Jones–Taylor–Thornton model) by MEGA7. Bootstrap analysis with 1000 replicates was performed to assess the statistical reliability of the tree topology.

### 4.3. Gene Cloning and Plasmid Construction

The full-length coding domain sequence (CDS) of *Bna.A07.WRKY70* (XP_ 013648025.1) without stop codon was amplified by the specific primer designed in NCBI (https://www.ncbi.nlm.nih.gov/tools/primerblast/, accessed on 20 October 2020). The total RNA was extracted from young leaves of the *B. napus* cultivar “ZS11” by the SteadyPure Plant RNA Extraction Kit (Accurate Biology, Changsha, China), and the RNA concentration was determined by spectrometry (Nano Drop; Thermo Scientific, Wilmington, MA, USA) (Supplementary Table S3) and quality was checked by 1% agarose gel electrophoresis. For cloning, first-stand cDNA was synthesized from total RNA using EasyScript One-Step gDNA Removal and cDNA Synthesis SuperMix (TransGen, Beijing, China). The CDS of *Bna.A07.WRKY70* was isolated from cDNA through PCR (Thermal Cycler Block, Thermo Fisher Scientific) using the high-fidelity thermostable DNA polymerase KOD-Plus-Neo (Toyobo Co., Ltd., Osaka, Japan). The PCR conditions were as follows: pre-denaturation at 94 °C for 2 min, followed by 35 cycles of 98 °C for 10 s, 55 °C for 30 s, and 68 °C for 1 min, and final extension at 68 °C for 7 min. Cloning primers are listed in Supplementary Table S1.

To construct the plasmid *35S:Bna.A07.WRKY70-GFP*, the CDS of *Bna.A07.WRKY70* without stop code was digested with the restriction endonucleases *XbaI* and *BamHI* and cloned into P1300-35S-green fluorescent protein (GFP) vector, which was driven by the CaMV35S (35S) promoter. Similarly, the digested PCR fragment of *Bna.A07.WRKY70* was also cloned into pGreen-35S-eGFP to produce a fusion of *GFP-Bna.A07.WRKY70* under the control of the 35S promoter. To obtain the construct of *pBna.A07.WRKY70:GUS*, the 2600 bp 5′ regulatory region upstream of the ATG start codon, as the *Bna.A07.WRKY70* promoter region was amplified and cloned into pHY107 [43]. The CDS of *Bna.A07.WRKY70* was cloned into the pGBKT7 vector containing the GAL4 DNA binding domain to form a construct of *pBD-Bna.A07.WRKY70*. Eight single colonies of each plasmid were selected randomly and sequenced by Sangon Biotechnology (Shanghai, China). Primers used for plasmid construction are listed in Supplementary Table S1.

### 4.4. Subcellular Localization of Bna.A07.WRKY70-GFP Protein

The *35S:GFP-Bna.A07.WRKY70* construct was transformed into *Agrobacterium tumefaciens* strain GV3101 and transiently expressed in the leaves of transgenic tobacco (*Nicotiana benthamiana*) carrying a nuclear localization signal as previously described [44]. Images of fluorescent signals were detected through a confocal laser scanning microscope (Leica TCS SP8 SR, Wetzlar, Germany) 72 h after agroinfiltration of the tobacco plants.

### 4.5. Transcriptional Activation Assays in Yeast

The construct of *pBD-Bna.A07.WRKY70* and the negative control pGBKT7 vector were transformed separately into the yeast strain Y2HGold, including the *HIS3* and *ADE2* reporter genes. The transformed strains were cultured on synthetic dropout nutrient medium without tryptophan (SD/-Trp) plates and then were spotted on SDO (SD/-Trp) and TDO (SD/-Trp/-His/-Ade) plates by diluting to different concentrations. The transcription activation activity of each construct was observed according to the growth conditions of the corresponding yeast cells after incubating for 2–3 days in a 30 °C incubator.

### 4.6. Generation of A. thaliana Transgenic Plants

The construct of *pBna.A07.WRKY70:GUS* and *35S:Bna.A07.WRKY70-GFP* was transformed into *Agrobacterium tumefaciens* strain GV3101, which was subsequently used to transform the *A. thaliana* wild type and *wrky70* mutant plants using the floral dip method [45]. The transgenic lines of *pBna.A07.WRKY70:GUS* in wild type was selected on soil using Basta^®^ and the transgenic lines of *35S:Bna.A07.WRKY70-GFP* in *wrky70* mutants were screened by hygromycin. All the transgenic plants were genotyped according to DNA and RNA analyses and selfed until T_3_ generation homozygous plants, which were generated and used for subsequent experiments.

### 4.7. RNA Extraction and RT-qPCR Analysis

The total RNA from various tissues of *B. napus* and leaves of *A. thaliana* were extracted using the SteadyPure Plant RNA Extraction Kit (Accurate Biology, Changsha, China). The quality of RNA was assessed using 1% agarose gel electrophoresis, and the concentration was determined by spectrometry (Nano Drop; Thermo Scientific, Wilmington, MA, USA) (Supplementary Table S3). RNA was reverse transcribed by EasyScript One-Step gDNA Removal and cDNA Synthesis SuperMix (TransGen, Beijing, China) according to the manufacturer’s instructions, and conditions were 37 °C for 15 min; 85 °C for 5 s, followed by maintaining at 4 °C. Quantitative real-time PCR (RT-qPCR) was utilized to evaluate gene expression with SYBR Green Master Mix (Cofitt, Hongkong, China) using the QuantStudio^TM^ 7 Flex Real-Time PCR System (Thermo Scientific), which were performed by three independent biological replicates with two technical replicates for each biological replicate. Reactions were performed in a total volume of 20 μL containing 100 nM of each primer and 2 μL of diluted cDNA (50 ng/μL) templates and amplified using the following cycling conditions: 95 °C for 2 min, 40 cycles of 95 °C for 15 s, 60 °C for 30 s, and 72 °C for 30 s. *AtACTIN7* (amplified product with 161 bp) and *BnACTIN7* (amplified product with 400 bp) were used as the internal control in *Arabidopsis* and rapeseed, respectively. For each reaction run, the specificity of the amplification was validated, and the threshold cycle (Ct) above the background was calculated using Bio-Rad iCycler (Bio-Rad, Hercules, CA, USA). The relative expression levels of the target genes were calculated using a modified double delta method [46]. Primers used for RT-qPCR analyses are listed in Supplementary Table S2.

### 4.8. Phenotypic Observation of A. thaliana Leaves

The seeds of the Col-0, the *wrky70* mutant, and three independent lines—*wrky70 35S:Bna.A07.WRKY70-GFP #4*, *#6*, *and #12*—were germinated on 1/2 MS agar medium for one week. Subsequently, the seedlings were transplanted into 8 × 8 cm pots. When the *A. thaliana* plants grew 35 days after germination (DAG), the phenotype of leaf senescence was observed and photographed by a camera (D7500, Nikon, Tokyo, Japan).

### 4.9. Measurement of the Chlorophyll Content

The fifth and sixth rosette leaf samples of Col-0, *wrky70* mutants, and *Bna.A07.WRKY70* transgenic plants from the fourth, fifth, sixth, and seventh weeks were separately collected and weighed and then placed in a 1.5 mL centrifuge tube with 1 mL extraction solution (80% acetone), soaked the leaves in the dark for 24 h until they faded [47]. To calculate the chlorophyll content of leaves, the 0.2 mL supernatant was absorbed into Costar 96 Flat Transparent plate, and the absorbance values at 663 nm and 645 nm were measured using a microplate reader (Infinite M200pro, Tecan, Mannedorf, Switzerland). Each experiment was represented by three biological replicates.

## 5. Conclusions

As an indispensable portion, leaf senescence spans the latter half of leaf development, which is essential to guarantee better production and survival of the next generation. This study suggested that *Bna.A07.WRKY70* may act as a negative regulator to share a conserved function with *AtWRKY70* in controlling leaf senescence when it is expressed in *A. thaliana*. Thus, *Bna.A07.WRKY70* can be utilized as a potential target to genetically manipulate leaf senescence and to create new stay-green materials to improve the rapeseed yield.

## Data Availability

All data included in this study are available upon reasonable request by contact with the corresponding author.

## Supplementary Materials

The following supporting information can be downloaded at: https://www.mdpi.com/article/10.3390/plants12020347/s1, Figure S1: Percent identity of full-length protein sequences of the WRKY70 protein from *A. thaliana* and *B. napus*; Table S1: Primers used for gene cloning and various constructs in the present study; Table S2: Primers used for RT-qPCR analysis in the present study; Table S3: The amount of RNA per sample in the *Arabidopsis thaliana* and *Brassica napus*.
