# Supplementary material for: Brassica napus Transcription Factor Bna.A07.WRKY70 Negatively Regulates Leaf Senescence in Arabidopsis thaliana
Source: Plants (Basel). 2023 Jan 11;12(2):347. doi: 10.3390/plants12020347 (PMC9867431; doi:10.3390/plants12020347)
Supplement: Supplementary file 1 [file plants-12-00347-s001.zip › plants-2130097-Supplementary Materials.pdf]

	AtWRKY70	Bna.A07.WRKY70	Bna.C06.WRKY70	Bna.A04.WRKY70	Bna.C08.WRKY70	Bna.A09.WRKY70	Bna.C04.WRKY70
AtWRKY70							
Bna.A07.WRKY70	66.01						
Bna.C06.WRKY70	65.02	86.41					
Bna.A04.WRKY70	63.82	73.46	73.46				
Bna.C08.WRKY70	63.37	71.52	71.52	69.90			
Bna.A09.WRKY70	62.71	70.87	70.87	68.61	84.14		
Bna.C04.WRKY70	61.97	71.52	71.20	86.73	67.64	66.34	

Percent identity of full-length protein

Figure S1. Percent identity of full-length protein sequences of the WRKY70 protein from *A. thaliana* and *B. napus*. AtWRKY70 (NP_191199.1), Bna.A07.WRKY70 (BnaA07G0195100ZS), Bna.C06.WRKY70 (BnaC06G0198900ZS), Bna.A04.WRKY70 (BnaA04G0035900ZS), Bna.C08.WRKY70 (BnaC08G0362900ZS), Bna.A09.WRKY70 (BnaA09G0519800ZS) and Bna.C04.WRKY70 (BnaC04G0308100ZS) proteins, performed by DNAMAN 8.

Supplementary Table S1. Primers used for gene cloning and various constructs in the present study.

Primer name	Primer sequence (5′–3′)	Annotation
Bna.A07.WRKY70-GFP-XbaI-F	GAGAACACGGGGGACtctagaATTCACCAAAATGGATATTGCTTGT	35S: Bna.A07.WRKY70-GFP construct
Bna.A07.WRKY70-GFP-BamHI-R	GCGACCGGTACCCGGggatccAACCGAAAACTGCTGCCAAC	
GFP-Bna.A07.WRKY70-HindIII-F	GTCGACGGTATCGATaagcttATGGATATTGCTTGTAATAAC	35S:GFP-Bna.A07.WRKY70 construct
GFP-Bna.A07.WRKY70-EcoRI-R	TCCCCCGGGCTGCAGgaattcAACCGAAAACTGCTGCCAAC	
pBna.A07.WRKY70:GUS-XmaI-F	ATCGAATTCCTGCAGcccgggGTTGTCGACTGGAGGTACGG	pBna.A07.WRKY70:GUS
pBna.A07.WRKY70:GUS-BamHI-R	CTGTCTAGAACTAGTggatccAGTAAGCGAGAGAAGGTGAAACT	
BD-Bna.A07.WRKY70-NdeI-F	TCAGAGGAGGACCTGcatatgATGGATATTGCTTGTAATAAC	BD-Bna.A07.WRKY70
BD-Bna.A07.WRKY70-EcoRI-R	TCGACGGATCCCCGGgaattcAACCGAAAACTGCTGCCAAC	
35S-F	GTCACTTTATTGTGAAGATAGTGG	It combines with Bna.A07.WRKY70-GFP-BamHI-R primer to genotype the corresponding construct
PGP2-R	CCTTATCGGGAAACTACTCACAC	It combines with GFP-Bna.A07.WRKY70-HindIII-F primer to genotype the corresponding construct
GUS103-R	ATCGTTAAAACTGCCTGGCAC	It combines with p Bna.A07.WRKY70:GUS-XmaI-F primer to genotype the corresponding construct
BD-F	TAATACGACTCACTATAGGGCGA	It combines with BD-Bna.A07.WRKY70-EcoRI-R primer to genotype the corresponding construct

Supplementary Table S2. Primers used for RT-qPCR analysis in the present study.

Primer name	Primer sequences (5′–3′)	Annotation
CAB1-F	CCAGAGGCATTCGCTGAGTTG	RT-qPCR analysis of *AtCAB1*
CAB1-R	CCTTACCAGTGACGATGGCTTG	
SAG13-F	AGGAAAACTCAACATCCTCGTC	RT-qPCR analysis of *AtSAG13*
SAG13-R	GCTGACTCGAGATTTGTAGCC	
SEN1-F	GTCATCGGCTATTTCTCCACCT	RT-qPCR analysis of *AtSEN1*
SEN1-R	GTTGTCGTTGCTTTCCTCCATC	
Bna.A07.WRKY70-F	ATGGATATTGCTTGTAATAAC	RT-qPCR analysis of *Bna.A07.WRKY70*
Bna.A07.WRKY70-R	AACCGAAAACTGCTGCCAAC	
AtACTIN7-F	GCCCCTGAGGAGCACCCAGTT	Internal control
AtACTIN7-R	CCGGTTGTACGACCACTGGCA	
BnACTIN7-F	TGGTTGGGATGGGTCAAAAAGA	
BnACTIN7-R	CGGAGGATAGCGTGAGGAAGAG	

Supplementary Table S3. The amount of RNA per sample in the *Arabidopsis thaliana* and *Brassica napus*.

Specie	RNA sample	Concentration (ng/µl)
Brassica napus	Roots	92.7
	Steams	118.2
	Leaves	204.5
	Flowers	89.8
	Developing seeds	82.9
Arabidopsis thaliana	Col-0	161.9
	wrky70	80.6
	wrky70 35S:Bna.A07.WRKY70-GFP #2	164.7
	wrky70 35S:Bna.A07.WRKY70-GFP #4	142.5
	wrky70 35S:Bna.A07.WRKY70-GFP #6	174.2
	wrky70 35S:Bna.A07.WRKY70-GFP #12	104.3
	wrky70 35S:Bna.A07.WRKY70-GFP #16	98.0
